# Supplementary material for: Alzheimer's Disease Risk Factor APOE4 Exerts Dimorphic Effects on Female Bone
Source: Adv Sci (Weinh). 2026 Apr 13;13(28):e23511. doi: 10.1002/advs.202523511 (PMC13185828; doi:10.1002/advs.202523511)
Supplement: Supplementary file 2 — Supporting File 2: advs74733‐sup‐0002‐FigureS1.pptx. [file ADVS-13-e23511-s001.pptx]

## Slide 1
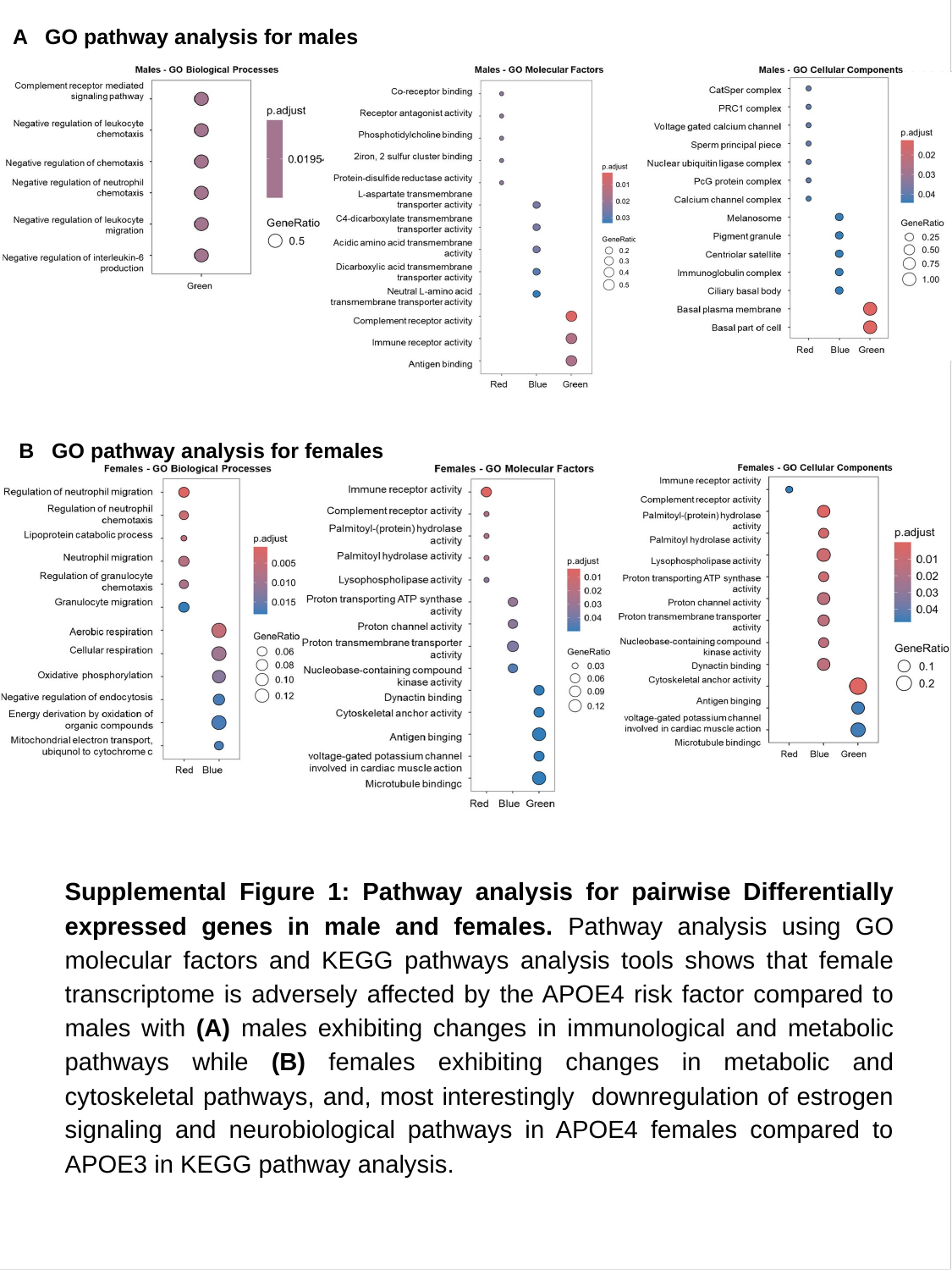

A GO pathway analysis for males
B GO pathway analysis for females
Supplemental Figure 1: Pathway analysis for pairwise Differentially expressed genes in male and females. Pathway analysis using GO molecular factors and KEGG pathways analysis tools shows that female transcriptome is adversely affected by the APOE4 risk factor compared to males with (A) males exhibiting changes in immunological and metabolic pathways while (B) females exhibiting changes in metabolic and cytoskeletal pathways, and, most interestingly downregulation of estrogen signaling and neurobiological pathways in APOE4 females compared to APOE3 in KEGG pathway analysis.
