# Supplementary material for: Alzheimer's Disease Risk Factor APOE4 Exerts Dimorphic Effects on Female Bone
Source: Adv Sci (Weinh). 2026 Apr 13;13(28):e23511. doi: 10.1002/advs.202523511 (PMC13185828; doi:10.1002/advs.202523511)
Supplement: Supplementary file 4 — Supporting File 4: advs74733‐sup‐0004‐FigureS3.pptx. [file ADVS-13-e23511-s005.pptx]

## Slide 1
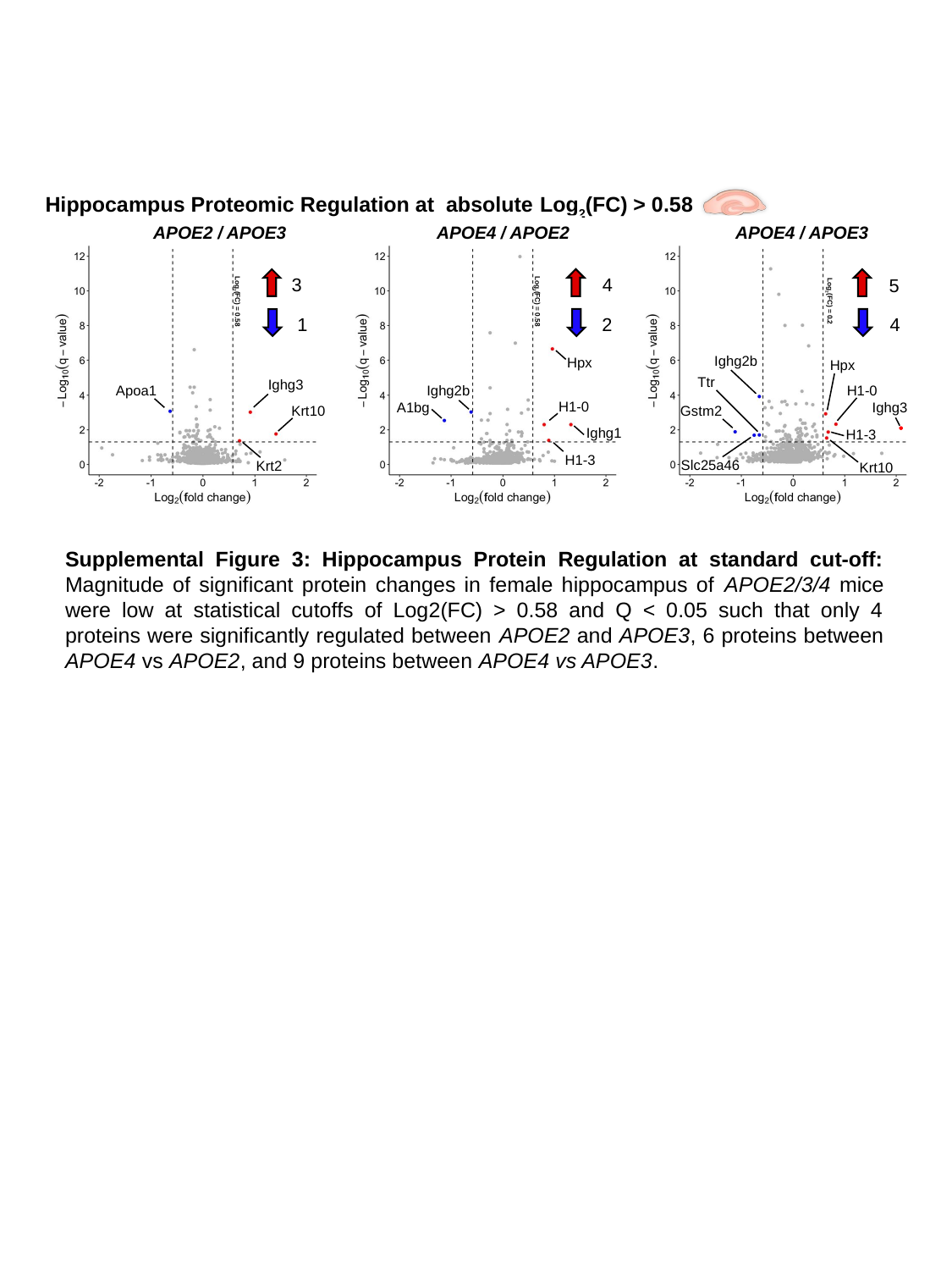

Hippocampus Proteomic Regulation at absolute Log2(FC) > 0.58
APOE2 / APOE3
3
1
Log2(FC) = 0.58
Ighg3
Apoa1
Krt10
Krt2
APOE4 / APOE2
4
2
Log2(FC) = 0.58
Hpx
Ighg2b
H1-0
A1bg
Ighg1
H1-3
APOE4 / APOE3
5
4
Log2(FC) = 0.2
Ighg2b
Hpx
Ttr
H1-0
Ighg3
Gstm2
H1-3
Slc25a46
Krt10
Supplemental Figure 3: Hippocampus Protein Regulation at standard cut-off: Magnitude of significant protein changes in female hippocampus of APOE2/3/4 mice were low at statistical cutoffs of Log2(FC) > 0.58 and Q < 0.05 such that only 4 proteins were significantly regulated between APOE2 and APOE3, 6 proteins between APOE4 vs APOE2, and 9 proteins between APOE4 vs APOE3.
