# Supplementary material for: Alzheimer's Disease Risk Factor APOE4 Exerts Dimorphic Effects on Female Bone
Source: Adv Sci (Weinh). 2026 Apr 13;13(28):e23511. doi: 10.1002/advs.202523511 (PMC13185828; doi:10.1002/advs.202523511)
Supplement: Supplementary file 5 — Supporting File 5: advs74733‐sup‐0005‐FigureS4.pptx. [file ADVS-13-e23511-s017.pptx]

## Slide 1
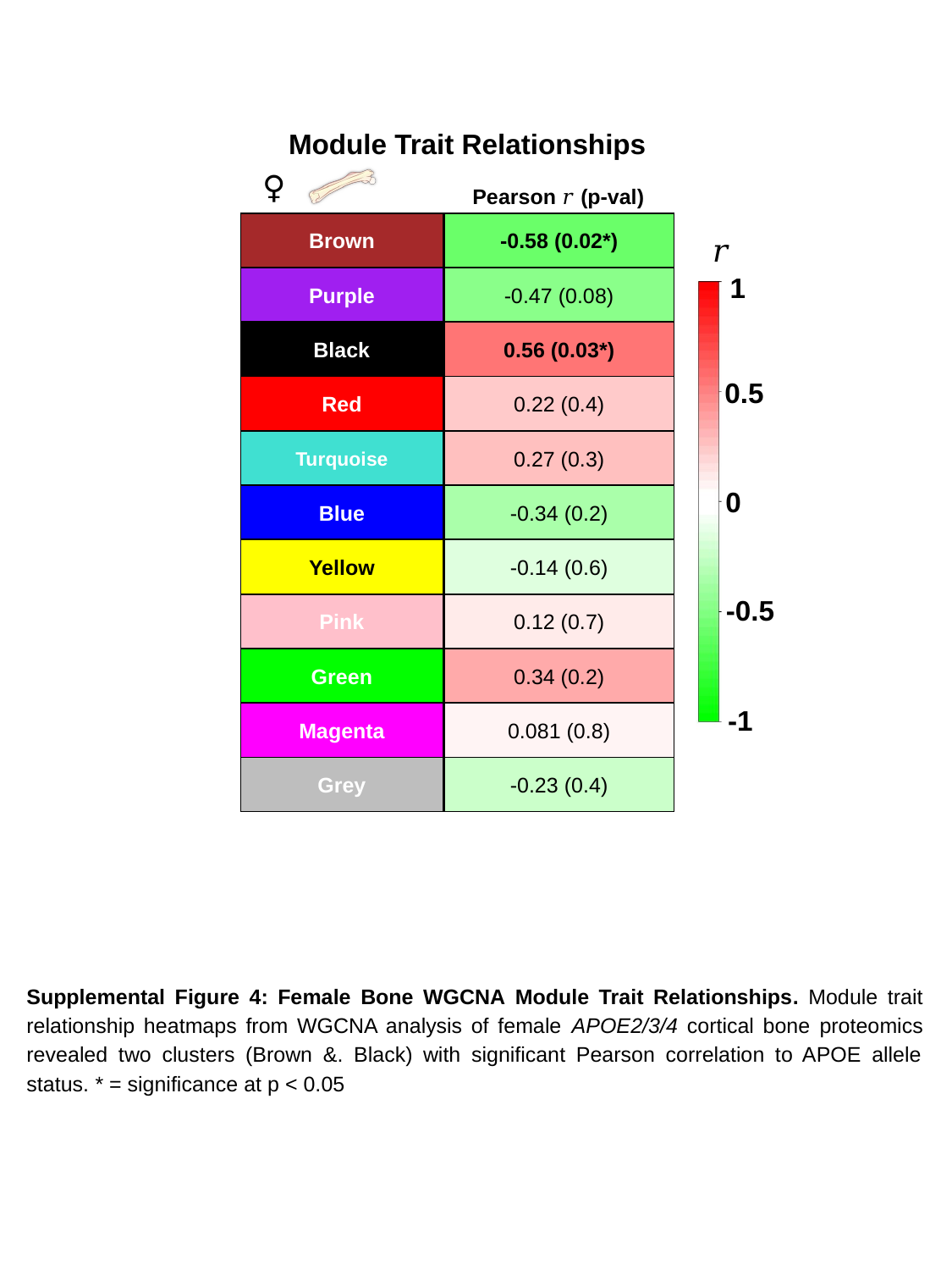

Module Trait Relationships
♀
Pearson 𝑟 (p-val)
Brown
-0.58 (0.02*)
𝑟
1
0.5
0
-0.5
-1
Purple
-0.47 (0.08)
Black
0.56 (0.03*)
Red
0.22 (0.4)
Turquoise
0.27 (0.3)
Blue
-0.34 (0.2)
Yellow
-0.14 (0.6)
Pink
0.12 (0.7)
Green
0.34 (0.2)
Magenta
0.081 (0.8)
Grey
-0.23 (0.4)
Supplemental Figure 4: Female Bone WGCNA Module Trait Relationships. Module trait relationship heatmaps from WGCNA analysis of female APOE2/3/4 cortical bone proteomics revealed two clusters (Brown &. Black) with significant Pearson correlation to APOE allele status. * = significance at p < 0.05
